# NEMA Performance Evaluation of CareMiBrain dedicated brain PET and Comparison with the whole-body and dedicated brain PET systems

**DOI:** 10.1038/s41598-019-51898-z

**Published:** 2019-10-29

**Authors:** Laura Moliner, Maria J. Rodríguez-Alvarez, Juan V. Catret, Antonio González, Víctor Ilisie, José M. Benlloch

**Affiliations:** 1 Instituto de Instrumentación para la Imagen Molecular (i3M), Universitat Politècnica de València – CSIC, Valencia, Spain; 2 Instituto de Automática e Informática Industrial (ai2), Universitat Politècnica de València, Valencia, Spain

**Keywords:** Molecular medicine, Imaging techniques

## Abstract

This article presents system performance studies of the CareMiBrain dedicated brain PET according to NEMA NU 2-2012 (for whole-body PETs) and NU 4-2008 (for preclinical PETs). This scanner is based on monolithic LYSO crystals coupled to silicon photomultipliers. The results obtained for both protocols are compared with current commercial whole body PETs and dedicated brain PETs found in the literature. Spatial resolution, sensitivity, NECR and scatter-fraction are characterized with NEMA standards, as well as an image quality study. A customized image quality phantom is proposed as NEMA phantoms do not fulfil the necessities of dedicated brain PETs. The full-width half maximum of the radial/tangential/axial spatial resolution of CareMiBrain reconstructed with FBP at 10 and 100 mm from the system center were, respectively, 1.87/1.68/1.39 mm and 1.86/1.91/1.40 mm (NU 2-2012) and 1.58/1.45/1.40 mm and 1.64/1.66/1.44 mm (NU 4-2008). Peak NECR was 49 kcps@287 MBq with a scatter fraction of 48% using NU 2-2012 phantom. The sensitivity was 13.82 cps/kBq at the center of the FOV (NU 2-2012) and 10% (NU 4-2008). Contrast recovery coefficients for customizing image quality phantom were 0.73/0.78/1.14/1.01 for the 4.5/6/9/12 mm diameter rods. The performance characteristics of CareMiBrain are at the top of the current technologies for PET systems. Dedicated brain PET systems significantly improve spatial resolution and sensitivity, but present worse results in count rate measurements and scatter-fraction tests. As for the comparison of preclinical and clinical standards, the results obtained for solid and liquid sources were similar.

## Introduction

National Electrical Manufacturers Association (NEMA) NU 2-2007^1^ and NU 2-2012^2^ standards constitute a set of methods under specific conditions that allow estimating the performance evaluation and comparison of Positron Emission Tomograph (PET) scanners. NU 2-2007 is expressly intended for Whole Body PETs (WB-PET) and NU 2-2012 presents minor modifications of the previous protocol. The measurements performed by these standards are the spatial resolution, sensitivity, counting rate performance, accuracy (correction for count losses and randoms) and image quality (accuracy of attenuation and scatter corrections).

In this work, we present the performance evaluation of the dedicated brain PET CareMiBrain based on NU 2-2012 and NU 4-2008^3^ (dedicated to preclinical equipment). The reason for including the small animal standard is the reduced dimensions of the CareMiBrain scanner (260 mm of gantry). Finally, we compare the performance evaluation for the most used WB-PETs and dedicated brain PETs found in the literature that fulfills the NEMA procedures. Some of these studies used the standard NU-2007 and therefore it is also included in this study. Table 1 shows the phantoms used in each standard and the main differences between them. Detailed information can be found in^1–3^.

**Table 1:** NEMA Measurements performed in this study and its main differences.

Measurement	Phantom/Reconstruction Details			Differences
	NU 4-2008	NU 2-2007	NU 2-2012	
Spatial Resolution	Encapsulated ^22^Na Source. Reconstructed with FBP	Capillary filled with ^18^F. Reconstructed with FBP	Capillary filled with ^18^F. Reconstructed with FBP	- Different positions for all three protocols - 2012 admits alternative iterative algorithms but is mandatory FBP reporting
Scatter fraction, count losses, random coincidences measurements	Cylindrical polyethylene phantom. Different dimensions for mouse, rat and monkey	Cylindrical polyethylene phantom for whole body.	Cylindrical polyethylene phantom for whole body.	- Different phantom in 2008 - Different phantom positioning and different tolerances for the activity filling between 2007–2012
Sensitivity	Encapsulated ^22^Na Source	Capillary filled with ^18^F and different sleeves sizes phantom	Capillary filled with ^18^F and different sleeves sizes phantom	- Different phantom in 2008 - Slightly different parameter calculation and tolerances for the activity filling between 2007–2012.
Image Quality, accuracy of attenuation correction and scatter correction	Customized phantom that includes uniformity region, rods and cold rods (air/water). Filled with ^18^F	Customized phantom that includes: uniformity region, lung insert, and 6 spheres (hot regions). Filled with ^18^F	Customized phantom that includes: uniformity region, lung insert, and 6 spheres (hot regions). Filled with ^18^F	- Different phantom in 2008 - Different phantom positioning, acquisition time and different tolerances for the activity filling between 2007–2012
Accuracy: corrections for count losses and randoms	Not Included	The same phantom as the scatter fraction test	The same phantom as scatter fraction test	- Different phantom positioning and different tolerances for the activity filling between 2007–2012 - The reconstructed image values are compared to different references between 2007–2012

## Materials and Methods

**Figure 1:**
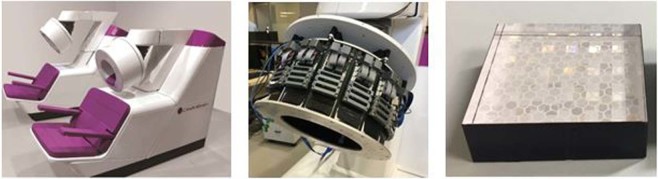
Image of the CareMiBrain system (left), detail of the detector electronics (center), crystal configuration (right).

CareMiBrain is a brain dedicated PET system developed by Oncovision S.A. (Spain). This device consists of three detector rings with 16 detector modules each forming a transaxial gantry of 256 mm and an axial length of 154 mm. Each detector module includes a 50 × 50 × 15 mm^3^ monolithic Lutetium Yttrium OrthoSilicate (LYSO) crystal (non-pixelated) coupled to a photosensor array of 12 × 12 Silicon PhotoMultiplier (SiPM) of the C-Series type from SensL (Cork, Ireland). All surfaces of the scintillation blocks are polished and the lateral ones are black-painted. The 50 × 50 mm^2^ entrance surface includes a retro-reflecting layer which bounces back the scintillation light to the point of emission. Coincidence timing window is 5 ns and the overall energy resolution is about 17%. The detectors have their own electronic board including the power supply, for both the SiPM and the electronics on the module (see Fig. 1).

CareMiBrain dimensions cover more than 95% of the patient’s percentile based on a 3D anthropometric database of the population framed in the INTERREG IVB SUDOE program^4^ carried out by the Biomechanics Institute of Valencia. During the scan, the patient is seated and the detector is placed surrounding the head (Fig. 1) leaving space for open eyes and visual external stimuli studies. The whole scanner footprint is 1.2 × 2.5 m^2^.

The most noticeable technological feature in comparison to other PET scanners is the use of a single monolithic crystal per module instead of small pixelated crystal arrays. Monolithic crystals together with the proprietary readout electronics have the advantage of a high resolution (1 mm) determination of the Depth Of Interaction (DOI) of the gamma rays inside the scintillation crystal thickness^5^. The (x, y) coordinates are obtained summing the 12 × 12 channels in columns and rows and applying the Raised to the Power (RTP) method^5^.

CareMiBrain works in 3D data acquisition mode. The transaxial Field of View (FOV) is defined by the number of modules in the detection ring and the acceptance angles of impinging photons. Coincidences among a detector and its nine opposite detector modules belonging to the same ring and the others are allowed during the acquisition. We define a pair detector as the two modules that can trigger an event. Therefore, the scanner has 648 possible pairs. Given the number of pairs, the number of Lines of Response (LOR) is determined by the virtual pixel dimension chosen. In this work, the pixels used are 2 mm × 2 mm, thus, we consider about 180 M LORs during the acquisition and reconstruction process.

Direct normalization method^6^ is applied to obtain a geometrical-free-artifact reconstructed image. To obtain the correction factors for normalization a ^22^Na cylindrical phantom with uniform activity (14.79 MBq) with a diameter and height of 240 mm and 150 mm respectively was, situated in the FOV and acquired during 20 hours.

### Spatial resolution

The spatial resolution of a system represents its ability to distinguish between two points after image reconstruction. The measurement of spatial resolution was performed according to both standards. For the preclinical standard a ^22^Na point source (a sphere with 0.3 mm diameter embedded in an acrylic cube of 10.0 mm) with an activity of 370 kBq (July 2011) was used. The source was located at the axial center of the FOV, and one-fourth of the axial FOV from the center of the axial FOV, at radial distances from the center of 0, 5, 10, 15, 25, 50, 75 and 100 mm. Each measurement took 300 seconds.

Following WB-PET PET standard performance, a capillary glass tube with ^18^F-FDG (FluoroDeoxyGlucose) was used, with an inner and outer diameter of 1 and 2 mm respectively. The starting activity was 121 kBq with a length of 1 mm inside the tube. The source was located at the axial center of the FOV, and three-eighths of the axial FOV from the center of the axial FOV, at 10 and 100 mm from the center in a radial direction. Each measurement took 300 seconds.

The experiments were repeated thrice. The acquisitions were reconstructed using 2D-Filtered BackProjection algorithm (FBP)^7^ with Single Slice ReBining method (SSRB)^6^, using an energy window of 30% and were analyzed according to their NEMA standards.

### Scatter fraction and count rate measurements

The objective of these measurements is to obtain system performance curves. These curves are the result of the analysis of the acquisitions in which the data are sorted into sinograms by SSRB, and processed to obtain an estimation of the true and random-scattered coincidences. With this aim and assuring certain conditions, several measures must be taken starting with an activity that guarantees the saturation of the detector until the detection of radiation is negligible. The Scatter Fraction (SF) estimation is performed with low activity rates ensuring the absence of random coincidences.

**Figure 2:**
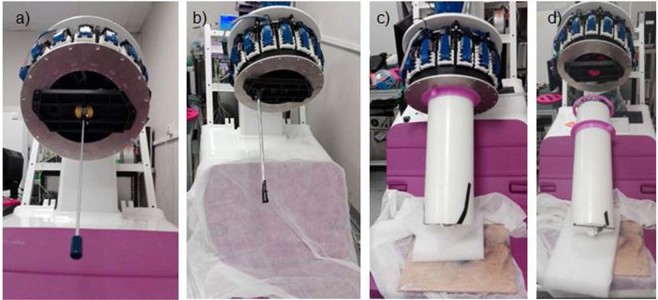
(**a**) Centered sensitivity NU 2-2012 phantom. (**b**) Phantom displaced 100 mm off the central axis. (**c**) Count rate performance measurement. (**d**) Image quality test. The custom phantom is placed on the support inside the tomograph.

In our case, scatter fraction and count rates tests were performed following WB-PET specifications (Fig. 2c). The phantom described in this standard was placed in the center of the tomograph employing support specifically designed for this purpose, achieving the same position as indicated for WB-PET devices.

In our case, each measurement lasted 100 seconds, with a delay time of 300 seconds in between acquisitions. The initial activity of the line source was 602.5 MBq. The SF estimation following NEMA indications was performed with acquisitions in the range of 7.4–14.8 MBq. The energy window used was 50%.

### Sensitivity

Sensitivity is a measure that indicates how many true coincidence events have been detected for a given source. For this purpose, a source is placed along the axial axis in order to acquire data over the entire length of the scanner. These acquired data are processed according to the standards to obtain a sensitivity value, utilizing analysis of sinograms (in case of NU 4-2008) or data extrapolation (in case of NU 2-2012).

According to NU 4-2008, the same solid source as in spatial resolution section is used to perform this measurement. In our case, the source was placed at the center of the transaxial FOV and moved along it in steps of 2 mm. The duration of each acquisition was 180 seconds.

According to NU 2-2012, we used the sensitivity phantom described in the standard. 600 seconds scans were taken with the 5 sets of aluminium sleeves with increasing thickness. The phantom was suspended in the center of the transaxial FOV, aligned with the axis of the tomograph. This measurement was repeated at 100 mm off the central axis (Fig. 2a,b). A 700 mm length plastic tube was used and filled with water mixed with ^18^F-FDG with an initial activity of 12.56 MBq for the center test and 7.16 MBq for the off-center test.

Two energy windows were considered: 355 to 664 keV (30%) and 255 to 765 keV (50%) for both standards.

### Image quality, the accuracy of attenuation and scatter corrections

The main objective of image quality testing is the measurement of Recovery Coefficients (RC). The recovery coefficients evaluate the system ability to discern hot or cold lesions contained in a radioactive background, giving an idea of the reconstructed image quality taking into account corrections for scattering, random counts, lost counts, positron range and partial volume effect. The spill-over ratio (SOR) is the ratio of the mean in each cold region and the mean of the hot uniform area.

**Figure 3:**
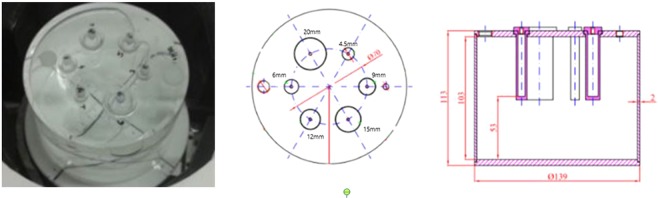
Image quality phantom.

WB-PET standard uses a torso-like phantom, while the preclinical standard has its own phantom. Dedicated tomographs have a very specific application and NEMA phantoms are not the most appropriate. With this motivation, we designed a custom phantom, with dimensions closer to a human head; 135 mm diameter and 103 mm height. Inside, six independent cylindrical rods with 50 mm height and diameters of 20, 15, 12, 9, 6 and 4.5 mm are placed (Fig. 3). The phantom designed is able to evaluate the recovery coefficients from 4.5 mm to 20 mm within a radioactive background covering the range of spatial resolutions provided by WB-PET scanners reported in the literature. The values of RC, SOR and their standard deviations were calculated based on the procedure described in NU 4-2008.

For the measurements, the containing cylinder was filled with 5.3 kBq/ml of ^18^F-FDG and the four small rods were filled at a 4:1 ratio. The rod of 15 mm was filled with non-radioactive water while the 20 mm rod contained only air. The acquisition lasted 1200 seconds and was processed using an energy window of 30%. To simulate the activity outside of the FOV, the count rate phantom was placed on the sofa with a linear source of 116 MBq (Fig. 2d). Finally, a reconstructed image of a patient from Hospital Clinico San Carlos from Madrid (Spain) is shown in this work. The acquisition took 660 seconds and the activity injected at the moment of the acquisition was 123 MBq.

The acquisitions were reconstructed using List Mode Ordered Subsets algorithm (LMOS)^8^ with 3 iterations, 12 subsets, voxel size of 1 × 1 × 1 mm^3^ and virtual crystal pixel size of 2 × 2 mm^2^. The scatter correction is based on the dual-energy window method^9^, whereas the random correction follows the single rate method^10^. The attenuation correction is performed using an attenuation map generated by segmentation of the reconstructed image^11^. In the case of the phantom, two different materials were considered for attenuation map: air and tissue. However, in the patient image, three attenuation materials were considered: air, bone and tissue. No further post-processing filters were applied.

### Compliance with ethical standards

The patient image shown is part of studies were all procedures performed are in accordance with the ethical standards of the Hospital Clinico San Carlos and in-agreement with the 1964 Helsinki declaration and its later amendments or comparable ethical standards.

The patient showed in Fig. 6 gave his informed consent to study participation.

## Results

### Spatial resolution

Tables 2, 3 and 4 collect the values obtained for spatial resolution. The difference between source sizes affects spatial resolution results.

**Table 2:** Spatial resolution (NEMA NU 4-2008).

Reconstructed image pixel size (mm): 0.25 mm Slice thickness (mm): 0.25 mm At axial center								
	5 mm		10 mm		15 mm		25 mm	
	FWHM	FWTM	FWHM	FWTM	FWHM	FWTM	FWHM	FWTM
Radial	1.51	2.75	1.58	2.87	1.64	2.98	1.52	2.78
Tangential	1.55	2.82	1.45	2.53	1.52	2.77	1.59	2.89
Axial	1.45	2.64	1.40	2.31	1.58	2.89	1.41	2.57
**At ¼ axial center**								
Radial	1.55	2.83	1.59	2.94	1.43	2.61	1.65	2.83
Tangential	1.59	2.89	1.58	2.88	1.55	2.82	1.67	2.85
Axial	1.45	2.64	1.42	2.60	1.37	2.50	1.42	2.60

**Table 3:** Extra spatial resolution values (NEMA NU 4-2008).

Reconstructed image pixel size (mm): 0.25 mm Slice thickness (mm): 0.25 mm At axial center								
	0 mm		50 mm		75 mm		100 mm	
	FWHM	FWTM	FWHM	FWTM	FWHM	FWTM	FWHM	FWTM
Radial	1.57	2.94	1.67	3.00	1.64	2.99	1.64	2.99
Tangential	1.53	2.90	1.51	2.75	1.76	3.02	1.66	3.21
Axial	1.36	2.62	1.44	2.63	1.44	2.63	1.44	2.63
**At ¼ axial center**								
Radial	1.56	2.85	1.80	3.22	1.70	3.09	1.85	3.34
Tangential	1.56	2.84	1.85	3.32	1.76	3.27	1.69	3.07
Axial	1.42	2.58	1.45	2.50	1.50	2.81	1.55	2.83

**Table 4:** Spatial resolution (NEMA NU 2-2012).

Reconstructed image pixel size (mm): 0.25 mm Slice thickness (mm): 0.25 mm At axial center				
	10 mm		100 mm	
	FWHM	FWTM	FWHM	FWTM
Radial	1.87	3.39	1.86	4.83
Tangential	1.68	3.07	1.91	3.43
Axial	1.39	2.54	1.40	2.56
**At 3/8 axial center**				
Radial	1.82	4.56	1.97	5.42
Tangential	1.77	3.23	1.85	3.38
Axial	1.37	2.50	1.45	2.64

### Scatter fraction and count rate measurements

**Figure 4:**
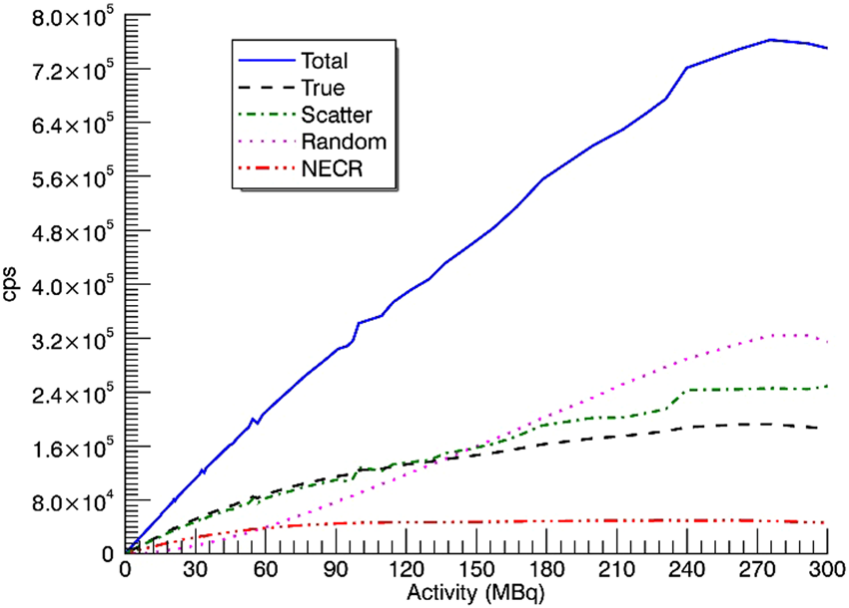
Count rate curves NU 2-2012.

Figure 4 shows the count rate curves as a function of the activity according to NU 2-2012 protocol. Noise Equivalent Count Rate measurement (NECR) peak achieved 49 kcps@287 MBq while the true peak was 193 kcps@287 MBq. The SF had a mean value of 48% for CareMiBrain system in a range of activities of 7.4–14.8 MBq.

### Sensitivity

**Figure 5:**
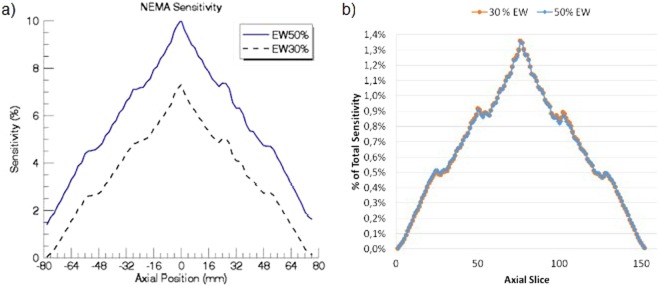
(**a**) Sensitivity according to preclinical standard (solid source). (**b**) Percentage of total activity acquired by each slice measured according to WB-PET standard in the center of the FOV.

The results for NU 4-2008 are shown in Fig. 5a. The sensitivity peak was 7% (for 30% of energy window) and 10% (for 50%). According to the NU 2-2012, the total sensitivity in the center was 13.82 cps/kBq and 11.05 cps/kBq (for 50% and 30% respectively), while 17.83 cps/kBq and 13.57 cps/kBq respectively at 100 mm-off-center as shown in Fig. 5b.

To compare both protocols, the average of the contributions of the ^22^Na source along the whole axial axis can be considered as an approximation of the sensitivity of a line source of 154 mm (5.69% and 3.59% for the 50% and 30% energy windows respectively). If we consider the liquid source homogeneous, the activity in the 154 mm FOV can be linearly estimated (*i*.*e*. 154/700 times the original activity). The values obtained with this estimation were similar: 1.25% vs. 1.38% for the 50% window and 0.79% vs. 1.10% for the 30% window. The difference between the measurements could be reduced with a higher sampling of the ^22^Na data, which would lead to a better approximation to a line source.

### Image quality, the accuracy of attenuation, and scatter corrections

**Figure 6:**
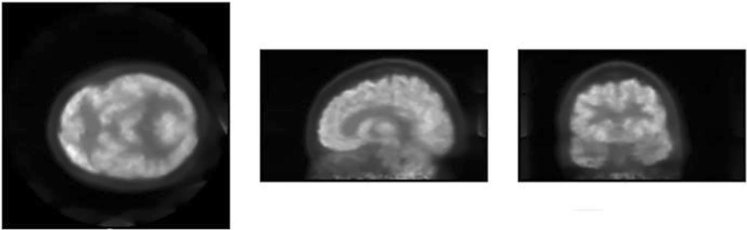
Patient image from Hospital Clínico San Carlos (Spain).

The results for the recovery coefficients were 0.73, 0.78, 1.14 and 1.01 (from smaller to bigger rod diameters) with standard deviations of 45–46% for all rods. The SOR were 0.002 and 0.0001 with standard deviations of 12.3% and 17% for air and water respectively. The patient image is shown in Fig. 6.

### Comparison with other tomographs

The most relevant characteristics of the brain dedicated and WB-PET systems are listed in Table 5, and NEMA results in Tables 6 and 7. There is no consensus on the image quality study for dedicated brain PETs and any work found in the literature present results for the NEMA “accuracy” section. These results for WB-PETs are given in Table 8, for completeness (procedures and phantoms are in NU 2-2007 and NU 2-2012).

**Table 5:** PET systems characteristics.

PET Name (company)	Type	Scintillator	Crystal Size (mm)	Sensor	aFOV (mm)	tFOV (mm)	NEMA standard
CareMiBrain (Oncovision)	Brain PET	LYSO	50 × 50 × 12	SiPM	154	240	2012 & 2008
BrainPET-4layers-MPPC-DOI^13^	Brain PET	4 layer LYSO	1.2 × 1.2 × 3, 4, 5 & 8	MPPC	201,6	330	2008 OR 2012
NeuroPET^14^	Brain PET\CT	LYSO:Ce	2.3 × 2.3 × 10	SiPM	220	250	2008 OR 2012
Human Brain Insert (Siemens)^15^	Brain PET Insert	LSO	2.5 × 2.5 × 20	APD	191	320	2007
G-PET^16^	Brain PET	GSO	4 × 4 × 10	PMT	256	300	2001 OR 1994
ECAT HRRT^17^	Brain PET	2 layers LSO/LYSO	2 × 2 × 10 & 10	PMT	230	320	2001
jPET-D4^18^	Brain PET	4 layers GSO	2.9 × 2.9 × 7.5	PS-PMT	312	260	2001
GAPD-PET^19^	Brain PET	LYSO	3 × 3 × 20	GAPD	60	390	2007 OR 2008
PET-HAT^20^	Brain PET	2layers GSO	4.9 × 4.9 × 7 & 8	PF-PMT	48	180	2001
MB-PET^*^ ^21^	Brain PET	LYSO:Ce	1 × 1 × 10	—	245	310	2008
MindView^22^	Brain PET Insert	LYSO	50 × 50 × 20	SiPM	154	220	NO NEMA
RF-penetrable PET insert^23^	Brain PET Insert	LYSO	3.2 × 3.2 × 20	SiPM	280	—	NO NEMA
Rainbow VHD (PINGSENG)^24^	Brain PET	LYSO:Ce	2.88 × 2.88 × 18	PMT	119	300	NO NEMA
HelmetPET^25^	Helmet brain PET	LYSO:Ce	1.5 × 1.5 × 10	MPPC	48	185	NO NEMA
SBPET^*^ ^26^	Spherical brain PET	Liquid Xenon	32 × 50 × 100	—	250	—	NO NEMA
Helmet Jaw PET^*^ ^27^	Helmet-Jaw PET	—	3 × 3 × 3	—	—	—	NO NEMA
Helmet-chin PET^*^ ^28^	Helmet-Chin PET	4 layers GSO	2.8 × 2.8 × 7.5	—	—	—	NO NEMA
Neuro-PET^29^	Brain PET	LYSO	3 × 3 × 20	SiPM	60	—	NO NEMA
Celestion (Toshiba)^30^	PET/CT	LYSO	4 × 4 × 4	PMT	196	700	2012
Biograph mCT flow (Siemens)^31^	PET/CT	LSO	4 × 4 × 20	PMT	221	700	2012
Biograph mCT (Siemens)^32^	PET/CT	LSO	4 × 4 × 20	PMT	221	700	2012
Biograph mMR (Siemens)^33^	PET-MR	LSO	4 × 4 × 20	PMT	258	588	2007
Vereo (Philips)^34^	PET/CT	LYSO	4 × 4 × 19	DPC	164	764	2012
Ingenuity TF (Philips)^35^	PET/CT	LYSO	4 × 4 × 22	PMT	180	256, 576, 676	2007
Ingenuity PET/MR (Philips)^36^	PET/MR	LYSO	4 × 4 × 22	PMT	180	675	2007
Geminity TF (Philips)^37^	PET/CT	LYSO	4 × 4 × 22	PMT	180	675	2007
SIGNA PET/MR (GE)^38^	PET/MR	LBS	4 × 5.3 × 25	SiPM	250	600	2012
Discovery MI (GE)^39^	PET/CT	LYSO	4 × 5.3 × 25	SiPM	200 mm	700 mm	2012
Discovery IQ (GE)^40^	PET/CT	BGO	6.3 × 6.3 × 30	PMT	200 mm	700 mm	2012

*Simulated devices.

**Table 6:** Spatial resolution (center axial FOV).

PET Name	Algorithm	Isotope	10 mm						100 mm					
			FWHM			FWTM			FWHM			FWTM		
			radial	tang.	axial	radial	tang.	axial	radial	tang.	axial	radial	tang.	axial
Celestion	SSRB + FBP	^18^F	4.5	4.7	4.4	<5.0	<5.1	<5.1	4.6	4.8	4.6	<5.4	<5.2	<5.1
Biograph mCT flow	FORE + FBP	^18^F	4.33	4.33	4.25	8.60	8.60	8.55	5.16	4.72	5.85	9.30	9.68	11.06
Biograph mCT	FORE + FBP	^18^F	5.0	5.0	6.4	10.8	10.8	11.8	4.9	4.9	5.7	9.3	9.3	10.7
Biograph mMR	FORE + FBP	^18^F	4.0	4.0	4.1	8.0	8.0	8.2	4.4	4.4	4.4	8.3	8.3	8.8
Vereos	3DFRP	^18^F	3.99	3.99	3.99	8.29	—	—	—	—	—	—	—	—
Ingenuity TF	3DFRP	^18^F	4.84	4.84	4.73	9.79	9.79	9.67	5.25	5.01	5.23	10.55	10.08	10.48
Ingenuity PET/MR	3DFRP	^18^F	4.7	4.7	4.6	9.4	9.4	9.5	5.0	5.3	5.0	9.9	10.5	9.7
Geminity		^18^F	5.06	4.84	4.73	9.7	9.7	9.6	5.03	4.89	5.2	10.3	10.2	9.6
SIGNA PET/MR	FBP	^18^F	4.4	4.10	5.34	—	—	—	5.78	4.44	6.74	—	—	—
Discovery MI	FBP	^18^F	4.02	3.97	4.39	8.52	8.19	10.12	5.28	4.23	5.63	9.95	8.83	11.80
Discovery IQ	OSEM (VPHD)	^18^F	4.2	4.7	4.8	9.5	9.8	11.2	5.6	5.1	4.8	11.4	10.2	11.1
**Dedicated PETs**														
CareMiBrain	SSRB + 2DFBP	^22^Na	1.72	1.66	1.71	3.13	3.02	3.11	2.65	2.20	1.74	5.90	6.90	4.80
CareMiBrain	SSRB + 2DFBP	^18^F	2.34	1.93	1.94	4.27	5.20	2.51	4.26	3.51	3.54	7.80	9.40	4.57
BrainPET-4layer MPPC	2DFBP	^22^Na	1.8–2.1	1.8–2.1	1.8–2.1	—	—	—	1.8–2.1	1.8–2.1	1.8–2.1	—	—	—
NeuroPET	FBP	^22^Na	3.2	3.2	3.5	6.0	6.0	6.8	5.2	3.1	4.0	6.8	4.8	6.2
Human Brain Insert	OP-3DOSEM	^18^F	1.8	2.9	2.7	5.8	7.0	11.0	3.6	6.0	4.4	5.7	11.5	9.5
G-PET	3D-FRP		4.2	4.2	5.2	10.0	10.0	8.2	5.0	5.0	6.0	12.2	12.2	10.2
ECAT HRRT	2D FBP	^18^F	2.6	2.7	3.0	—	—	—	3.0	3.1	5.1	—	—	—
jPET-D4	SSRB + 2DFBP	^18^F	3.1	3.1	3.1	—	—	—	3.7	3.5	3.1	—	—	—
GAPD-PET		^22^Na	3.0	3.0	—	—	—	—	4.6	3.3	—	—	—	—
PET-HAT	SSRB + 2DFBP	^22^Na	4.0	4.0	—	—	—	—	4.2	4.2	—	—	—	—
MB-PET	MLEM	^22^Na	1.02	1.21	1.27	—	—	—	1.28	1.41	2.05	—	—	—

**Table 7:** Count rate evaluation and sensitivity.

PET system	NECR Peak (kcps)	NECR [A] (kBq/ml)	True Peak (kcps)	True [A] (kBq/ml)	Scatter Fraction (%)	E. Window (keV)	Sens. Center (cps/kBq)	Sens. 100 mm (cps/kBq)
Celestion	70.	29.6	220	—	33	—	3.8	3.8
Biograph mCT flow	185.	29.0	634	42.4	33.5	—	9.6	9.6
Biograph mCT	186.	30.1	—	—	37.7 (NECR)	—	13.3	13.1
Biograph mMR	196.	24.4	—	—	37.9 (NECR)	—	10.0	10.0
Vereos	157.6	52.8	625	—	31.6	—	5.39	5.41
Ingenuity TF	124.	20.3	364.5	35.0	30.4	—	7.39	7.28
Ingenuity PET/MR	88.5	13.7	—	—	26	—	7.2	7.00
Geminity	112.	15 0.0	264.	16.52	24.7	—	7.9	7.9
SIGNA PET/MR	218.2	17.8	—	—	43.6	—	22.5	23.3
Discovery MI	201.1	22.1	875.9	35.4	40.4	—	13.4	14.0
Discovery IQ	123.	9.1	490.1	25.8	36	—	20.43	22.8
**Dedicated PETs**								
CareMiBrain	49	287 MBq	193	287 MBq	48	355–664	11.05	13.57
BrainPET-4L-MPPC	44.7	17.5 kBq	—	—	48.3	—	21.4	23.7
NeuroPET	22.7	2.9	—	—	—	—	11.6	13.9
Human Brain Insert	30.7	7.3	323	22.7	38–42	—	7.2%	7.9%
G-PET	60	7.40	132	13.69	30–46	—	4.79 (peak at 0.08)	—
ECAT HRRT	45	—	500	15	—	—	2.5% (absolute)	2.7% (absolute)
jPET-D4	82	8.7	—	—	42	400–600	19.3	—
GAPD-PET	—	—	—	—	—	—	—	—
PET-HAT	0.82	—	—	—	60	—	0.72% (^22^Na, center)	—
MB-PET	—	—	—	—	48	350–600	37 (total, ^22^Na)	—

**Table 8:** PET image quality (contrast recovery) for different sphere diameters (whole-body PETs).

PET Name	Contrast Recovery (Background Variability)						
	10 mm	13 mm	17 mm	22 mm	28 mm	37 mm	Relative count rate error at NECR-peak (%)
Celestion	27,9 (6,2)	48,6 (5,3)	52,0 (4,6)	60,5 (4,1)	72,9 (3,9)	77,1 (3,8)	17.3 (TOF-LM-OSEM)
Biograph mCT flow	28,5 (24,3)	42,3 (42,6)	58,4 (568,35)	71,7 (70,7)	70,1 (70,3)	78,3 (78,25)	3.7 (FORE-FBP)
Biograph mCT	37	66	72,2	77,3	81	85,3	1.9 (FORE-FBP)
Biograph mMR	23,4	57,1	75,2	83	58,8	67,5	4.9 (FPRE-FBP)
Vereos	38,4 (8.4)	61,3 (7.1)	65,9 (5,9)	68.5 (4,6)	83,4 (3,5)	86,4 (2,8)	—
Ingenuity TF	0,39	0,73	0,74	0,8	0,84	0,81	—
Ingenuity PET/MR	30 (7)	50 (6)	61 (4)	70 (3)	78 (3)	81 (3)	—
Geminity	34,70 (8,65)	51,43 (7.89)	60,83 (7.05)	72,13 (6.15)	73.88 (5.46)	77.65 (5.04)	
SIGNA PET/MR	35,2 (4,9)	48,9 (4,0)	59,9 (3,2)	68,6 (2.7)	79,2 (2.2)	87.4 (1.9)	3.3 (OSEM)
Discovery MI	51.7 (10)	61.5 (7.8)	66.2 (6.0)	81.3 (4.8)	86.6 (3.8)	90.0 (3.0)	2–3 (not in NECR-peak)
Discovery IQ	22 (3.3)	68 (2.0)	68 (2.0)	76 (2.5)	73 (2.5)	81 (2.5)	3.9 (OSEM)

## Discussion

Dedicated brain PETs present better results than WB-PETs in spatial resolution tests, CareMiBrain being the system that reports the best results. When both protocols are compared, the solid source results improve when compared with liquid sources (with larger dimensions). The use of ^22^Na solid source is easier, cost-effective (even ecological) compared to ^18^F liquid sources. For logistical reasons, we recommend a ^22^Na source. Since the results are sensitive to the reconstruction algorithm (2D-FBP, 3D-FBP, rebinning, filters, etc.) it could be interesting to perform this test free of algorithmic dependence *i*.*e*., Siddon-like-back-projection^12^ or just Full Width at Half Maximum (FWHM) measurements in the data sinograms.

Regarding count rate measurements, WB-PETs present a higher NECR peak and lower SF than dedicated brain PETs in all cases, but total count rates are comparable. The scatter detection is related to the system geometry, and smaller gantry translates into greater SF. The NECR curve depends (in the denominator) on the scatter, therefore, its peak is smaller for smaller gantry systems even if the total count rates are similar. The SF for CareMiBrain is in the range of other brain PET systems and NECR and trues peaks are overcome by jPET and G-PET. All systems have used NU 2-2012 phantom for the counting rate performance test and that is the main reason to use it in the present study. Figure 6 shows a patient image from CareMiBrain system, acquired at typical injection activity for a dedicated brain PET study, far below the NECR peak measured. Usually, dedicated PETs work at lower rates than the whole-body systems due to its proximity to the organ of study, allowing a higher frequency of patient monitoring.

Signa PET and Discovery IQ report the best results for the sensitivity test, and the brainPET 4 layers MPPC DOI, followed by jPET, NeuroPET and CareMiBrain present the best results for dedicated brain PETs. Finally, the values for the recovery coefficients showed an acceptable performance for attenuation and scatter corrections with the proposed measurements.

## Conclusion

Dedicated brain PET systems improve spatial resolution and sensitivity, but present worse results in count rate measurements and scatter fraction tests. However, the study should be re-performed for all the tomographs with a different phantom that appropriately adjusts the dimensions and characteristics of the brain to draw further conclusions for brain devices.

NEMA standards are an extremely useful tool for the comparison of different PET scanners, but, given the emerging dedicated brain PET systems, it could be interesting to redefine a standard exclusively for these tomographs. Comparing standards, the results are similar and we recommend the use of the solid sources due to logistic reasons.

In view of the results, performance characteristics of CareMiBrain system are at top of the current PET technologies.
